# Using next generation transcriptome sequencing to predict an ectomycorrhizal metabolome

**DOI:** 10.1186/1752-0509-5-70

**Published:** 2011-05-13

**Authors:** Peter E Larsen, Avinash Sreedasyam, Geetika Trivedi, Gopi K Podila, Leland J Cseke, Frank R Collart

**Affiliations:** 1 Biosciences Division, Argonne National Laboratory, Lemont, IL 60490, USA; 2 Department of Biological Sciences, University of Alabama in Huntsville, Huntsville, AL 35899, USA

## Abstract

**Background:**

Mycorrhizae, symbiotic interactions between soil fungi and tree roots, are ubiquitous in terrestrial ecosystems. The fungi contribute phosphorous, nitrogen and mobilized nutrients from organic matter in the soil and in return the fungus receives photosynthetically-derived carbohydrates. This union of plant and fungal metabolisms is the mycorrhizal metabolome. Understanding this symbiotic relationship at a molecular level provides important contributions to the understanding of forest ecosystems and global carbon cycling.

**Results:**

We generated next generation short-read transcriptomic sequencing data from fully-formed ectomycorrhizae between *Laccaria bicolor *and aspen (*Populus tremuloides*) roots. The transcriptomic data was used to identify statistically significantly expressed gene models using a bootstrap-style approach, and these expressed genes were mapped to specific metabolic pathways. Integration of expressed genes that code for metabolic enzymes and the set of expressed membrane transporters generates a predictive model of the ectomycorrhizal metabolome. The generated model of mycorrhizal metabolome predicts that the specific compounds glycine, glutamate, and allantoin are synthesized by *L. bicolor *and that these compounds or their metabolites may be used for the benefit of aspen in exchange for the photosynthetically-derived sugars fructose and glucose.

**Conclusions:**

The analysis illustrates an approach to generate testable biological hypotheses to investigate the complex molecular interactions that drive ectomycorrhizal symbiosis. These models are consistent with experimental environmental data and provide insight into the molecular exchange processes for organisms in this complex ecosystem. The method used here for predicting metabolomic models of mycorrhizal systems from deep RNA sequencing data can be generalized and is broadly applicable to transcriptomic data derived from complex systems.

## Background

Within days of germination, 95% of the short roots of most conifers and deciduous trees form ectomycorrhizae (ECM) with soil fungi [1], a form of symbiosis between plants and fungi whose evolution dates back 360-410 million years [2]. In ectomycorrhizae, the fungus forms a mycelial sheath around the plant's root, called the Hartig net that isolates the root from the soil and inhibits the development of short root hairs. Nutrients are exchanged between fungus and root across the apoplast, a zone that is outside both root and fungus, preventing direct contact between the fungus and plant cytoplasm and requiring that nutrients exchanged cross both fungal and plant cell walls. In ectomycorrhizae symbiosis, fungal and plant metabolisms are connected by a suite of transporters that shuttle essential nutrients across the apoplast from one organism to another. This union of the plant and fungal metabolisms, termed the mycorrhizal metabolome, provides greater environmental fitness to the partners in the symbiotic relationship than can be provided by either organism's metabolism alone. The mutualistic association provides the fungus with a source of photosynthetically derived carbohydrates [3]. As much as 25% of the plant's net photosynthesis is used to support its fungal partner [3,4]. The metabolic contributions of the fungus in return for those sugars are more diverse. The fungus increases the absorptive surface area of the root by the formation of an intense network of very thin hyphae in the soil, and is thereby able to explore and to access nutrients from a greater volume of soil than could be exploited by the plant's roots alone [5]. Ectomycorrhizae fungi also contribute to tree nutrition by the mobilization of nutrients from organic material in the soil [6] and through mineral weathering [7]. The network of hyphae in the soil is effective in taking up organic and inorganic nutrient resources such as phosphorus, nitrogen, zinc, copper and provides these nutrients to the host plant [8,9]. The symbiotic fungus also provides a higher tolerance against abiotic and biotic stresses such as drought, toxic heavy metal concentrations and protection from pathogens [5,10] and buffers the plant against sudden changes in its environment [11,12].

An experimental model for the mycorrhizal system uses the ectomycorrhizae fungus *Laccaria bicolor *and tree species *Populus tremuloides *(aspen). Not only do these organisms readily form mycorrhizae in the laboratory, but also the *L. bicolor *and the closely related species *Populus trichocarpa *genomes have been sequenced and annotated through the efforts of the Department of Energy and Joint Genome Institute's [13,14]. We used next generation sequencing (NGS) of the fully formed mycorrhizal transcriptome to construct a model of the ectomycorrhizal metabolome. The model is comprised of those expressed ectomycorrhizal genes that code for proteins that are either enzymes participating in metabolism or transporters capable of conveying nutrients from one organism to another. Previous studies reporting on differential expression [15-19] in mycorrhizal systems identify some aspects of regulation and metabolism but do not provide a comprehensive representation of the ectomycorrhizal metabolome. Although differential expression can highlight where a transcriptome differs in one biological condition to another, a full understanding of metabolism does not require that an associated gene or enzyme be differentially expressed to play a key role in a metabolic network, only that it be expressed. These previous studies have focused on discrete elements derived from either aspen or *L. bicolor *transcription profiles. Our approach simultaneously evaluates the transcript profiles of aspen and *L. bicolor *to identify the ways which fungal and plant metabolism merge to form this ecologically important symbiotic relationship. In the analysis of the predicted model of the ectomycorrhizal metabolome, we identify those regions in the model where aspen and *L. bicolor *expressed enzymatic activities that its partner does not. Where those unique metabolic contributions overlap with statistically significant, enriched expressed gene models for transporters, we find the likely nutrients that are exchanged between aspen and *L. bicolor *in the ectomycorrhizal interaction.

## Results

Ectomycorrhizae were established in Woody Plant Media (WPM) as an approach for identification of metabolic pathways unique to *L. bicolor *or aspen. This plant growth matrix is comprised only of nutrient salts and is devoid of any complex sources of organic nitrogen or phosphorus. Observations or inference for metabolism of complex molecules for ectomycorrhizae grown in this medium must therefore be attributable to the synthetic abilities of *L. bicolor *and aspen and would not be attributable to the symbiont catabolism of any compounds present in the media. *L. bicolor *and aspen roots were allowed to form fully mature ectomycorrhizae in culture, then were harvested and RNA extracted. The harvested mycorrhizal RNA is a mixture of transcripts from both *L. bicolor *and aspen. Using Illumina NGS technology and 'BowStrap' a tool for identifying statistically significant levels of gene expression, we sequenced the mycorrhizal transcriptome and identified the gene models that are detected as statistically significantly expressed at p < 0.001 in both biological replicates (Table S1). We make the assumption that if a gene is detected as expressed, then the protein encoded by that gene is also expressed. The predicted ectomycorrhizal metabolome was constructed by identification of enzymes and transporters associated with cellular metabolic compounds. Expressed genes that code for annotated enzymes were mapped to Kyoto Encyclopedia of Genes and Genomes (KEGG) metabolic pathways and expressed transporter proteins for sugars, phosphorous, and nitrogen containing compounds were identified and mapped to specific metabolites.

### Identification of expressed and enriched membrane transporters

In the ectomycorrhizal interaction, the individual enzymes and transporters of plant and fungal metabolism remain largely compartmentalized, prevented from directly interacting by the apoplastic space. To exchange nutrients, plant and fungus must have the capacity for importing and exporting nutrients across their cell walls. For the ectomycorrhizal metabolome, nitrogen, phosphorous and carbohydrate compounds are essential elements of nutrient exchange and Gene Ontology (GO) annotations [20] were used as a systematic approach to identify expressed genes in the mycorrhizal transcriptome annotated as members of these transporter classes. GO annotations for predicted proteins coded by best gene models from sequenced genomes were generated by automated BLAST homology to proteins of previously identified function [13]. These GO annotations are corroborated by individual publications on specific transporters for amino acids [21], ammonium [21,22], sugar [19], and inorganic phosphate [13] which have been identified as encoded in the genome.

Members of all the transporter classes are detected as expressed in the mycorrhizal transcriptome (Table 1). The *L. bicolor *genome encodes fewer transporters than does aspen in the selected analysis set but a larger proportion of the transporter genes is expressed. The expression patterns for the nitrogen and carbohydrate transporters in this study are similar to those reported using whole genome expression arrays [19,21,22]. A single inorganic phosphate transporter is annotated in the *L. bicolor *genome consistent with the previous observations of a limited number of fungal phosphate transporters in mycorrhizal symbiosis [23-25]. The sets of expressed mycorrhizal transporter genes were analyzed for significant enrichment of specific annotations. At a stringency of Cumulative Binomial Distribution (CBD) pVal < 0.05, expressed transporters are enriched for amino acid transport in *L. bicolor *and aspen (Table 1). Sugar porter activity is enriched only in *L. bicolor*. Ammonium transporters and inorganic phosphate transporters are not enriched in either the *L. bicolor *or aspen transcriptome.

**Table 1:** Expressed mycorrhizal transporters identified by their Gene Ontology annotation in either *L. bicolor *or aspen

Transporter*		Annotation		# Genomic	Expressed	pVal
Nitrogen	Amino acid transport	GO:0006865	*L. bicolor*	31	87.10%	0.038
			Aspen	125	72.80%	0.031
	Ammonium transporter activity	GO:0008519	*L. bicolor*	9	88.90%	0.116
			Aspen	17	52.90%	0.567

Carbon	Sugar porter activity	GO:0005351	*L. bicolor*	27	92.60%	0.023
			Aspen	110	61.80%	0.372

Phosphorus	Inorganic phosphate transporter activity	GO:0005315	*L. bicolor*	1	100.00%	0.13
			Aspen	13	53.80%	0.519

Transporters are categorized as "Nitrogen", "Carbon", and "Phosphorus" using GO annotation associated with the indicated metabolic compounds transported in the mycorrhizal metabolome. "# Genomic" indicates the total number of gene models with a specific GO annotation in the aspen or *L. bicolor *genome. "Expressed" is the percent of expressed vs. total transporter genes for the indicated organism in the mycorrhizal transcriptome. "pVal" is the CBD-pValue for the enrichment of a transporter annotation in the set of "Expressed" relative to the "# Genomic".

The lack of enrichment for ammonium transporter genes suggests that ammonium is not one of the primary mediums of interaction between aspen and *L. bicolor *in spite of the fact that ammonium salts are present in the growth medium. Amino acid transport, enriched in both aspen and *L. bicolor *suggests that amino acids are a major metabolite that is shared during ECM interaction. A number of nitrogen containing compound such as ammonia and amino acids have been proposed as candidate compounds for nitrogen exchange in ECM systems [26-28]. The correlation of the present model with amino acid transport is consistent with experimental data for arbuscular fungal and plant systems [29]. The transported amino acids are likely originated from the action of fungal enzymes as the growth medium lacks amino acids. Aspen transcriptome is not enriched for sugar transporters, but aspen does not require them. Sucrose molecules enter the apoplast from aspen roots via diffusion, where it is hydrolyzed into hexose sugars by a sugar invertase produced by aspen [30]. The glucose and fructose products of the invertase reaction are substrates for specific fungal hexose transporters [19]. The observed enrichment of sugar transporters in the *L. bicolor *transcriptome is consistent with the experimental data demonstrating transfer of carbohydrates across the apoplast interface in ectomycorrhizal roots of aspen [31]. The hexose transporter subset of enriched sugar transporters identified in this analysis overlaps with an expression profiling and characterization analysis of hexose transporters proteins encoded in the genome of *L. bicolor *[19].

### Model of the Mycorrhizal Metabolome

**Figure 1:**
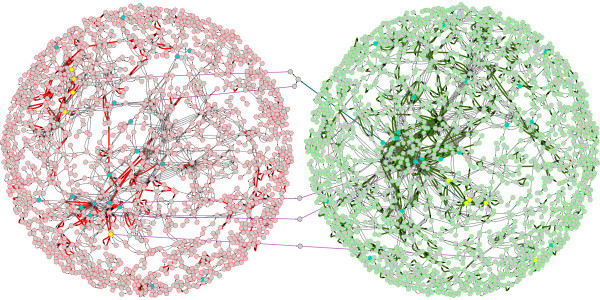
**Mycorrhizal Metabolome**. Solid lines represent enzyme functions identified in annotated transcriptomes, dashed lines represent predicted transfer of metabolites across cell membranes by diffusion (teal lines) or active transport (purple lines) and nodes represent predicted metabolites, inferred by the reactions catalyzed by expressed genes annotated with specific enzyme functions. Nodes bordered in green are predicted for aspen metabolism, nodes bordered in red are predicted for *L. bicolor *metabolism, and nodes bordered in gray represent metabolites transported across apoplastic space. Edges are highlighted red or green if the represented enzyme function is uniquely expressed by either *L. bicolor *or aspen respectively. Nodes representing metabolic compounds are highlighted yellow are sugar compounds predicted to be transported by the set of statistically enriched sugar porters in *L. bicolor*. Nodes highlighted in blue are amino acids, likely transported by the set of statistically enriched amino acid transporters in both *L. bicolor *and aspen. This figure was generated using Cytoscape v2.6.1 [67]. The network in this figure is available for download as Additional File 1, Data S1.

The mycorrhizal metabolome map depicts all the metabolic compounds inferred by expressed genes annotated with enzyme functions (Figure 1). The respective metabolomes of these organisms are connected by a suite of transporters that shuttle essential nutrients across the apoplastic space. Potential gateways for linkage of these compartmentalized *L. bicolor *and aspen metabolisms were identified using the amino acid and sugar transporters enriched in the set of mycorrhizal expressed genes. The predicted mycorrhizal metabolome is comprised of 3472 metabolic compounds connected by 5156 enzyme reactions. Reactions are mediated by 948 specific enzyme functions, of which 168 enzyme functions are uniquely expressed by aspen and 85 enzyme functions are uniquely expressed by *L. bicolor*.

### Mapping aspen expressed genes to metabolic pathways in KEGG

**Figure 2:**
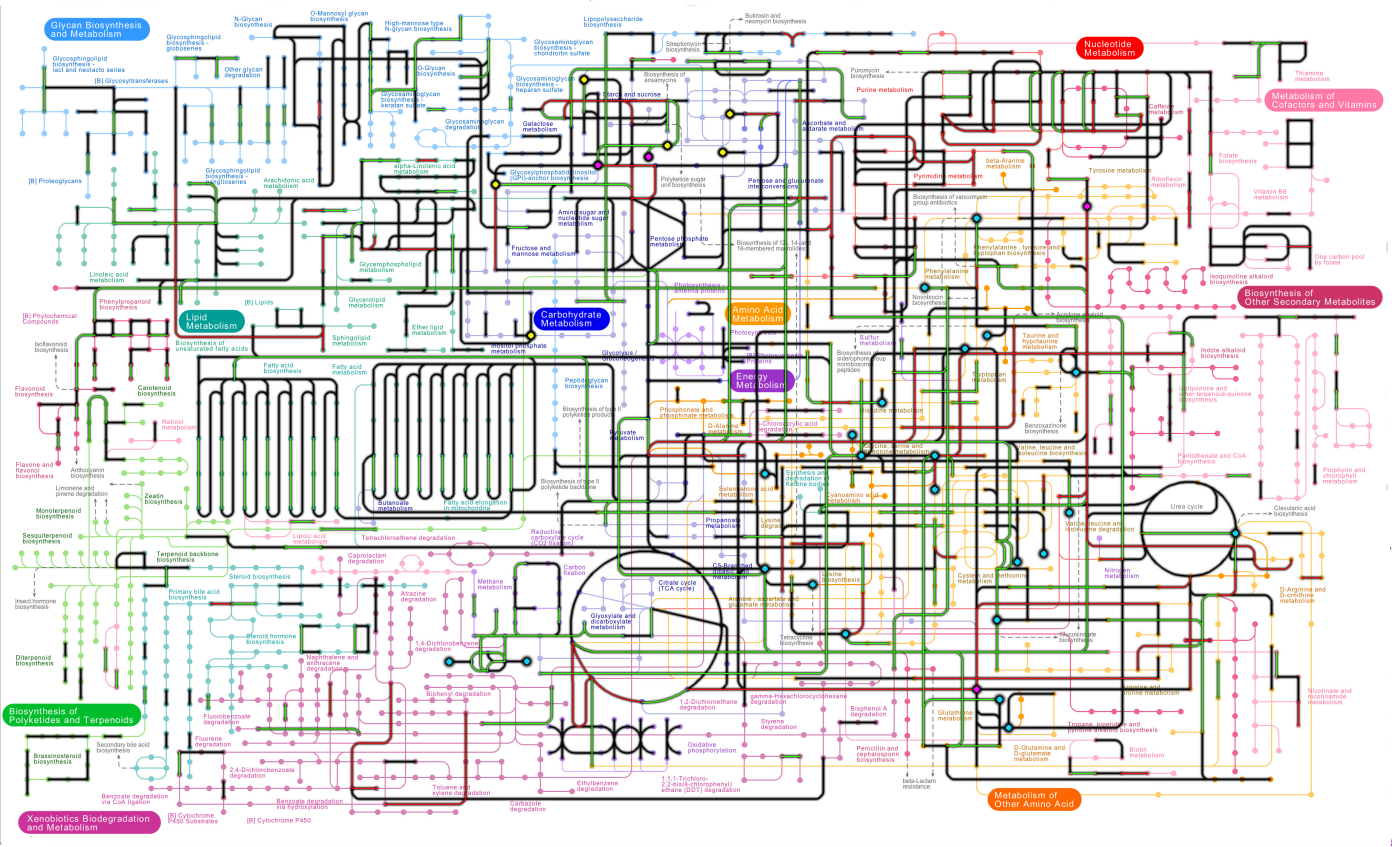
**Mycorrhizal metabolome mapped onto KEGG Global metabolism**. A subset of the Mycorrhizal Metabolome was generated by mapping expressed enzyme functions onto KEGG Global Map (map01100). Nodes in this figure are metabolic compounds. Edges are enzymatic transformations. Highlighted edges indicate that an EC activity has been detected in mycorrhizal transcriptome. An edge highlighted with red indicates an expressed gene annotated with an EC activity unique to the *L. bicolor *transcriptomes. Green highlighted edges are unique to the aspen transcriptome. Nodes highlighted in yellow are sugar compounds predicted to be transported by the set of statistically enriched sugar porters in *L. bicolor*. Nodes highlighted in blue are amino acids, likely transporter by the set of statistically enriched amino acid transporters in both *L. bicolor *and aspen. Nodes highlighted in pink are compounds that are likely to be metabolites directly shared between *L. bicolor *and aspen metabolism during mycorrhizal interaction. An interactive and much higher resolution version of this figure can be found at: http://www.bio.anl.gov/molecular_and_systems_biology/MycorMetabolome/Supplementalfigures.htm

To better visualize the predicted Mycorrhizal Metabolome, reactions in the complete mycorrhizal transcriptome were mapped onto KEGG Global metabolism (map01100). Global Metabolism map is comprised of 145 metabolic sub-networks [32]. This map is an expert-curated metabolic pathway that highlights an organism's core metabolic pathways and is a useful tool for visualizing key features out of the much more inclusive set of all possible metabolic interactions in the complete mycorrhizal metabolome. The significantly expressed genes of the mycorrhizal transcriptome that code for proteins annotated with EC activities were mapped to the global KEGG Metabolism Map and generated a sub-set of enzymatic reactions predicted to take place in aspen- *L. bicolor *mycorrhizal system (Figure 2). In this illustration, the nodes correspond to metabolic compounds while the edges represent enzymatic transformations of the compounds. Although many unique enzyme functions are shared in both organisms, expressed enzyme functions unique to the aspen or *L. bicolor *transcriptome are indicated by green or red edge colors, respectively. Of all enzyme reactions represented in KEGG global metabolism map01100, 30% are expressed in complete mycorrhizal transcriptome, 25.2% in aspen transcriptome, and 21.5% in the *L. bicolor *transcriptome. Of the 145 KEGG subnetworks that make up global metabolism KEGG map01100, 58.6% in the complete mycorrhizal transcriptome, 31.7% in the *L. bicolor *transcriptome, and 46.0% in the aspen root transcriptome are detected with at least 30% of enzyme activities significantly expressed.

(An interactive version of Figure 2, allowing a user to zoom in on any of the 145 metabolic sub-networks of map01100, is available in Supplementary Data, Figure S1-a http://www.bio.anl.gov/molecular_and_systems_biology/MycorMetabolome/Supplementalfigures.htm.

### Expressed mycorrhizal enzymes include core metabolic activities

The utility of the derived metabolic network (Figure 1) as a model for metabolism during mycorrhizal interaction was evaluated by analysis expressed sequences for core metabolic functions. The ability of the sequence data to fully represent expression of core metabolic functions would indicate the suitability of the transcriptomic data to investigate all predicted metabolic pathways. The approach and results are largely consistent with a previous transcript profiling study that delineated the major pathways of carbohydrate metabolism in *L. bicolor *[33]. To determine if genes that are expected to be expressed in aspen and *L. bicolor *are detected as expressed, we consider enrichment of core metabolism KEGG maps in the observed transcriptomics data. Expressed enzyme activities in the KEGG pathways for the core metabolic pathways pyruvate metabolism, glycolysis/gluconeogenesis (map00010), citrate cycle (map000230), Fatty acid metabolism (map00071), purine metabolism (map00230), and pyrimidine metabolism (map00240) are significantly enriched (p-value less than 0.0005) in both aspen and *L. bicolor *transcriptomes.

Many enzyme activities that are expressed in common between aspen and *L*. bicolor should also illuminate core metabolic activities. Metabolic pathways significantly enriched for enzyme activities expressed in both organisms (Table 2) are largely composed of core metabolic pathways. Examples of KEGG pathways for carbohydrate metabolism; glycolysis (KEGG map00010) and citrate cycle (map00020), energy metabolism; oxidative phosphorylation (map00190), nucleotide metabolism including purine (map00230) and pyrimidine (map22240) metabolism, and amino acid metabolism make up the majority of significantly shared KEGG pathways for shared and expressed mycorrhizal enzyme activities. This makes intuitive sense, as the basal metabolism required for living things should be well represented in both aspen and *L. bicolor *transcriptomes. Of more direct relevance in understanding the unique aspects of this interaction are the common signaling pathways enriched for activities expressed in both aspen and *L. bicolor *during mycorrhizal symbiosis. Phosphatidylinositol signaling system (map04070) and calcium signaling pathway (map040200) have 100% overlapping annotated expressed enzyme activities between aspen and *L. bicolor*. Calcium has been demonstrated to be an important intracellular signal involved in the regulation of fungal-plant symbiosis gene expression [16,18]. Regulation of calcium levels is an early signaling event in many plant/microbe interactions and sequential elevations of cytoplasmic and nuclear calcium levels may have a regulatory role in symbiosis [34]. The phosphatidylinositol signaling system is related to plant development and regulation, such as root hair growth [35]. This mechanism is relevant to ectomycorrhizal interactions, in which plant root hair growth is specifically suppressed in the formation of mycorrhizae by *L. bicolor*. The enrichment of activities for these common signaling pathways is consistent with commonalities observed for modulation of host cell responses in symbioses [36,37].

**Table 2:** Metabolic pathways significantly enriched for enzyme activities expressed in both aspen and *L. bicolor*

KEGG Class	KEGG Pathway (map#)	% Shared annotations	pVal
Amino Acid Metabolism	Alanine and aspartate metabolism (00252)	70.00%	6.79E-03
	Arginine and proline metabolism (00330)	85.70%	2.01E-03
	Glutamate metabolism (00251)	70.00%	6.79E-03
	Valine, leucine and isoleucine degradation (00280)	76.50%	4.01E-03

Biosynthesis of Polyketides and Terpenoids	Diterpenoid biosynthesis (00904)	100.00%	6.62E-03

Carbohydrate Metabolism	Butanoate metabolism (00650)	70.60%	9.72E-03
	Citrate cycle (TCA cycle) (00020)	72.20%	6.49E-03
	Glycolysis/Gluconeogenesis (00010)	70.40%	2.24E-03
	Pentose phosphate pathway (00030)	68.20%	6.99E-03
	Starch and sucrose metabolism (00500)	66.70%	3.38E-03

Energy Metabolism	Oxidative phosphorylation (00190)	90.00%	3.85E-03

Lipid Metabolism	Ether lipid metabolism (00565)	88.90%	5.95E-03
	Fatty acid metabolism (00071)	80.00%	3.59E-03
	Glycerolipid metabolism (00561)	93.80%	2.93E-04
	Glycerophospholipid metabolism (00564)	69.60%	4.74E-03
	Sphingolipid metabolism (00600)	75.00%	6.06E-03

Nucleotide Metabolism	Purine metabolism (00230)	61.10%	1.34E-03
	Pyrimidine metabolism (00240)	65.50%	4.90E-03

Signal Transduction	Calcium signaling pathway (04020)	100.00%	6.62E-03
	Phosphatidylinositol signaling system (04070)	100.00%	1.83E-03

Translation	Aminoacyl-tRNA biosynthesis (00970)	95.50%	2.30E-05

The "% Shared annotations" represent the percent of shared relative to the total annotations for the indicated pathway.

### Identification of unique metabolic capabilities

The expressed activities mapped to KEGG pathways were analyzed to identify metabolic activities that are uniquely expressed only in aspen or *L. bicolor*. The KEGG Pathways enriched for enzyme activities unique to *L. bicolor *(Table 3) consists of only two pathways. Both amino sugar and nucleotide sugar metabolism (map00520) and arginine and proline metabolism (map00330) pathways are significantly enriched for expressed *L. bicolor *enzymes. The pathways associated with the arginine and proline metabolism (map00330) are extensive and both Aspen and *L. bicolor *have unique and enriched enzyme activities that map to this pathway. The transcriptomic data from *L. bicolor *is consistent with the observation that the mycorrhizal transcriptome is significantly enriched for amino acid transporters encoded by both aspen and *L. bicolor*. The unique metabolic activities expressed in *L. bicolor *mediate metabolism of glutamate, arginine, proline, and aspartate (illustrated in Additional File 1, figure S1-f, S1-g, S1-h, and S1-i).

**Table 3:** Expressed mycorrhizal EC activities unique to *L. bicolor *mapped to enriched KEGG pathways

KEGG Class	KEGG Pathway (map#)	Unique	pVal
Amino Acid Metabolism	Arginine and proline metabolism (00330)	27.6%	5.20E-03
Carbohydrate Metabolism	Aminosugars metabolism (00530)	30.0%	5.56E-04

Another set of unique activities in *L. bicolor *is the capability for the synthesis and utilization of allantoin via ureidoglycolate or urea (illustrated in Additional File 1,figure S1-b and S1-c). This capability is represented by the uric acid oxidase, allantoinase, allantoicase, and urease proteins encoded in the *L. bicolor *genome and expressed in the mycorrhizal metabolome. The assigned enzyme activities and annotations are supported by sequence alignments to conserved domain database profiles (CDD of NCBI; http://www.ncbi.nlm.nih.gov/Structure/cdd/cdd.shtml), for these enzymes [38]. The *L. bicolor *urate oxidase protein sequence (EC 1.7.3.3, Protein ID 190858) generates a position-specific scoring matrix (PSSM) alignment to the conserved domain for urate oxidase (d00445) with and E-value of 2.03e-61. The *L. bicolor *protein sequences for allantoinase (EC3.5.2.5), allantoicase (EC3.5.3.4), and urease (EC3.5.1.5) generate similar PSSM alignments consistent with the assigned activities (allantoinase,TIGR03178, E-value of 8.08e-121; allantoicase, TIGR02961, E-value of 4.71e-99; and urease TIGR01792, E-value of 0e+00. These observations suggest that the main nutrients *L. bicolor *exchanges with aspen roots for sugars are nitrogen-based compounds more complex than the ammonium salts already present in the media [26,27].

Pathways enriched for enzyme activities unique to aspen (Table 4) are organized into several general categories. The first and largest category is the set of "KEGG Plant" metabolic pathways. Flavonoid biosynthesis (map00941), phenylpropanoid biosynthesis (map00940), and carbon fixation in photosynthetic organisms (map00710) are plant-specific pathways in KEGG that should be expressed in aspen and absent in *L. bicolor*. Those enzyme activities expressed in aspen but not *L. bicolor *that map to biosynthesis of steroids (map00100), share considerable overlap with the pathway for synthesis of plant hormones (map01070) suggesting this observation is generally consistent with the category of plant-specific metabolism. The KEGG pathways for phenylalanine metabolism (map00360), methionine metabolism (map00271), and arginine and proline metabolism (map00330) can be grouped into a category of nitrogen metabolism. DDT degradation (map00351) is a xenobiotic degradation KEGG pathway and may be involved in the metabolism of signaling molecules synthesized by *L. bicolor *during mycorrhizal interaction. The KEGG pathway group for carbohydrate metabolic networks uniquely expressed in aspen during mycorrhizal interaction and includes starch and sucrose metabolism (map00500) and pyruvate metabolism (map00620). Unique carbohydrate metabolism present in aspen but absent in *L. bicolor *conforms to our understanding of the biological basis for the ECM interaction. *L. bicolor *is poorly suited for metabolizing complex sugars from its environment and is largely dependent upon its plant symbiotic partner to provide hexoses for its survival. A closer look at the specific unique activities expressed in aspen supports this interpretation. One of the unique activities possessed by aspen is the capacity to break down starch and generate fructose and glucose from sucrose. Aspen also expressed a capacity for metabolism of plant cell wall components pectin and pectate that is absent in *L. bicolor *transcriptome. These integral reactions of the starch and sucrose metabolism KEGG pathway (Additional File 1, Figure S1) represent prototypical plant pathways and additionally specific EC activities supportive for mycorrhizal symbiosis.

**Table 4:** Expressed mycorrhizal EC activities unique to aspen, mapped to enriched KEGG pathways

KEGG Class	KEGG Pathway (map#)	Unique	pVal
Amino Acid Metabolism	Methionine metabolism (00271)	40.30%	4.13E-04
	Phenylalanine metabolism (00360)	72.50%	1.57E-14
	Arginine and proline metabolism (00330)	37.50%	4.29E-03

Carbohydrate Metabolism	Pyruvate metabolism (00620)	33.30%	3.19E-03
	Starch and sucrose metabolism (00500)	47.60%	0.00

KEGG plant	Flavonoid biosynthesis (00941)	71.40%	1.54E-14
	Carbon fixation in photosynthetic organisms (00710)	31.30%	6.87E-03
	Phenylpropanoid biosynthesis (00940)	26.80%	2.98E-03

Lipid Metabolism	Biosynthesis of steroids (00100)	49.30%	1.22E-06
	Fatty acid biosynthesis (00061)	38.50%	8.14E-04

Xenobiotics Biodegradation and Metabolism	DDT degradation (00351)	100.00%	1.64E-12

## Discussion

### The predicted mycorrhizal metabolome

Transcriptome data mapped at the level of KEGG pathways enabled identification of unique metabolic capabilities contributed by aspen and *L. bicolor *to the predicted mycorrhizal metabolome. To identify *a priori *the specific molecular mechanisms of those contributions it is necessary to consider the mycorrhizal metabolome model as a complete network of compartmentalized metabolisms interacting by transported metabolites through expressed membrane transporters. The process is enabled by identification of regions in the model represented in Figure 1 that exhibit unique interconnecting enzyme activities. Particular emphasis is assigned to those regions of unique activities that are also associated with compounds predicted to be transported by expressed, enriched amino acid transporters or sugar porters. Those identified regions underscore the most likely metabolic compounds that are synthesized by one of the ECM partners and transported across the apoplast to the partner organism. Several regions are identified using this strategy and lead to the identification of five compounds; fructose, sucrose, glycine, glutamate, and allantoin (highlighted in pink in Figure 1 and in interactive Additional File 1, Figure S1). These compounds meet the three criteria imposed for analysis of the predicted mycorrhizal metabolome; 1) the activities are uniquely expressed in one of the organisms, 2) the synthesized compound can be matched to appropriate exchange transporters, and 3) there is a reciprocal pathway in the partner organisms to enable utilization of the synthesized compound.

#### L. bicolor imports fructose and glucose hydrolyzed from sucrose by aspen

In ectomycorrhizal symbiosis, sucrose is released into the apoplast where it may be hydrolyzed into hexose sugars by a plant-provided acid hydrolase, EC3.2.1.26 [30,39]. Five out of the eight aspen genes models annotated with the activity EC3.2.1.26 are significantly expressed in mycorrhizae (illustrated in Additional File 1, Figure S1-d and S1-e). However, no gene in *L. bicolor *is annotated with this activity indicating the fungus is dependent upon aspen's ability to cleave sucrose into hexoses that can be imported by *L. bicolor*. This hypothesis from the predicted mycorrhizal metabolome is consistent with accepted models for sugar transfer and with experimental observations [30]. The genome of *L. bicolor *encodes several hexose importer genes [19] which are expressed in mycorrhizae (Table 1).

#### L. bicolor synthesizes glycine and glutamate for import by aspen

In spite of the presence of abundant ammonium salts in the growth media, we observe that *L. bicolor *is expressing enzymes for synthesizing nitrogenous compounds and membrane transporters for their export to aspen. Aspen expresses the transporters necessary for uptake and metabolism of those amino acids. The ability to absorb amino acids through the roots appears ubiquitous in plant species [27,40,41]. Glutamic acid in particular has been demonstrated to be significantly absorbed through the root systems [42] and glycine uptake has been observed in trees [41,43,44]. Unique enzymatic transformations expressed in *L. bicolor *generate L-glutamate from ammonium via EC 1.4.1.2, glutamic dehydrogenase. Aspen expressed a unique enzymatic pathway that converts L-glutamte to succinyl-CoA (Additional File 1, Figure S1). Aspen expresses a unique pathway from glycine to glycolysis/glucogenesis (illustrated in Additional File 1, Figure S1-f and S1-g).

#### L. bicolor possess capability for synthesis and utilization of allantoin

Another set of unique activities in *L. bicolor *is the capability for the synthesis and utilization of allantoin via ureidoglycolate or urea (illustrated in Additional File 1, Figures S1-b and S1-c). Allantoin is used by plants, bacteria, and some fungi as a nitrogen and carbon source [45] and is a candidate metabolic product that is useful to aspen. The expression of these enzymes in *L. bicolor *indicates compounds derived from this pathway may supplement the ability of the fungus to supply nitrogen to the plant. Examples of plants that are able to import allantoin through their roots and the ability of some plants to use allantoin as sole nitrogen source have been previously reported [46,47]. There is evidence that allantoin transporters are expressed in mycorrhizae. *L. bicolor *possesses at least one gene annotated as an allantoin permease, 248955, which is highly homologous to yeast protein DAL4, allantoin permease (1293888) with a BLASTp eValue 2e-88. Aspen also has at least one allantoin uptake transporter gene, 421134, homologous to Arabidopsis allantoin uptake ATUPS5 (NM_202186.2) with a BLASTp eValue of 8e-166. The current predicted metabolome model does not indicate the utilization of allantoin by aspen roots. This is, however, the result that is expected. Allantoin has been previously identified as one of the primary nitrogenous compounds exported from soybean root nodules to be metabolized in shoots [48,49]. Assuming that allantoin is used similarly in aspen, that the metabolic pathways for immediate utilization of allantoin should not be observed as expressed in the root, but should be detected in transcriptomic analysis of aspen shoots..

## Conclusions

We have generated a predicted model of *L. bicolor *and aspen root mycorrhizal metabolome using transcriptomic data. The mycorrhizal metabolome is comprised of the expressed metabolic enzymes in the mycorrhizal transcriptome and the transporters required for the exchange of metabolic compounds. In mycorrhizal symbiosis, aspen exchanges with *L. bicolor *photosynthetic sugars for nutrients. This expectation is validated by the statistically enriched expression of sugar porters by *L. bicolor*. The enrichment of KEGG amino acid metabolism pathways with unique expressed enzyme activities and the enrichment for expressed amino acid transporters for both aspen and *L. bicolor *indicate that, for mycorrhizae formed in WPM, *L. bicolor*'s debt to aspen for carbon is paid with organic nitrogen. *L. bicolor *expresses the metabolic capacity to synthesize nitrogenous compounds such as glycine, glutamate and allantoin, via pathways not expressed in aspen roots. In the growth conditions used here, the predicted exchange compounds are the fructose and glucose as well as organic nitrogen compounds, specifically glycine, glutamate and perhaps allantoin. The predictions suggest *L. bicolor *is an active metabolic partner in addition to passively extending the absorptive surface of aspen roots. This role encompasses uptake of ammonium from the medium and synthesis of more complex compounds provided to the plant. Additional experiments in different nutrient environments are expected to uncover additional mechanisms of mycorrhizal metabolic interactions.

The model we generated confirms prior biological knowledge and predicts previously unobserved mechanisms of mycorrhizal interaction. To generate the model required transcriptomic analysis in addition to knowledge of aspen and *L. bicolor *genomic annotations as it is necessary to know not only the complete metabolic capacity of an organism, but what fraction of that capacity is being actively transcribed under specific biological conditions or developmental state. These predictions are suitable for the design of future, hypothesis driven biological experiments. A deepening understanding of the molecular components of mycorrhizal interactions, an important component of terrestrial forest ecosystems, will have applications in global carbon management and sustainability. The approach applied to this ectomycorrhizal system can also be generalized to additional symbiotic metabolomic systems. This method depends on accurate annotation of genomes and a complete picture of metabolism from KEGG. In the JGI gene model annotations for *L. bicolor *version 1, nearly 60% of the predicted gene models do have annotation and 30% do not share homology to any previously identified protein. As the quality of gene model annotation continues to improve and as more complete metabolic pathways are published, generation of future metabolomic models will become more and more accurate. Our available and interactive model of the mycorrhizal metabolome will serve as an important resource for other investigators.

## Methods

### Laboratory mycorrhizal cultures of *L. bicolor *and aspen seedlings

Mycorrhizal aspen and *L. bicolor *were grown in biological duplicates. *L. bicolor *(Maire) Orton (strain S238N) culture was maintained on Modified Melin Norkan's (MMN) media as described [50] at 20°C. *Populus tremuloides *seeds were surface sterilized and germinated on McCown's woody plant media (WPM) in Petri dishes as described [51]. One week old germinated seedlings were transferred to Magenta vessels (Sigma, St. Louis, MO) containing the interaction medium (WPM with 1.5% of sucrose). The seedlings were grown under 16 hr light and 8 hr dark cycles at 24°C for 4-5 weeks until fine lateral roots were developed. *L. bicolor *mycelial plugs were transferred to Magenta vessels with aspen plants for ectomycorrhizae formation. Mycorrhizal roots were visualized approximately after 6 weeks. Mycorrhizal roots were collected, snap frozen in liquid nitrogen and stored at -80°C. Total RNA was extracted from mycorrhizal samples by CTAB method [52] and RNA quality was assessed by gel electrophoresis prior to library preparation. Total RNA was treated with RQ-DNase (Promega, Madison, WI) to remove any traces of DNA. The transcriptomic sequence was derived from the two independent biological samples but the data sets were combined for this analysis. Analysis of the independent set indicated 94% of aspen reads detected as expressed in one replicate were detected in the other. Significance of overlap for aspen is a p-value less than 1e-256. 95% of *L. bicolor *reads detected as expressed in one replicate were detected in the other. Significance for overlap for *L. bicolor *is a p-value less than 1e-256.

### mRNA-Seq sample preparation

Procedures described for preparation of mRNA for the mouse transcriptome analysis [53] were used with some modifications. Ten ug of total RNA from each sample was hybridized to Sera-mag oligo (dT) beads (Thermo Scientific) for mRNA purification. Purified mRNA was fragmented by addition of 5X fragmentation buffer (Illumina, Hayward, CA) and was heated for 5 min at 94° C in a thermocycler. First strand cDNA was synthesized using random primers to eliminate the general bias towards 3' end of the transcript. Second strand cDNA synthesis was done by adding GEX second strand buffer (Illumina, Hayward, CA), dNTPs, RNaseH and DNA Polymerase I followed by incubation for 2.5 h at 16° C. Second strand cDNA was further subjected to end repair, A-tailing, and adapter ligation in accordance with the manufacturer supplied protocols. Purified cDNA templates were enriched by PCR amplification with Phusion DNA polymerase (Illumina, Hayward, CA) and the samples were cleaned using QIAquick PCR purification columns and eluted in 30 μl EB buffer as per manufacturer's instructions (QIAGEN, CA). Purified cDNA libraries were quantified using Nanodrop spectrophotometer and loaded onto Illumina flow cells with one of the flow cells reserved for a recurrent internal standard as control for sequencing efficiency. Over 25 million sequence reads were generated for analysis.

### Detecting significant gene model expression

To detect significant gene model expression in collected transcriptomics data, we used the application 'BowStrap'. 'BowStrap' is a bootstrap style application of the available short sequence-aligning program Bowtie. As not every generated sequence read that does align can be uniquely aligned to a single location in a gene model, BowStrap takes into account those short sequence reads that align to more than one gene model location and calculated gene expression as number of aligned RNAseq Reads per Kilobase per Million reads (RRKM) with an associated standard error. The ultra-fast sequence alignment program "Bowtie-0.9.9" [54] was used to generate alignments of sequence reads to genomes and gene models. Bowtie uses a Burrows-Wheeler index to rapidly align sequences to a pre-processed indexed set of sequences. When Bowtie encounters a short sequence that does not align to a single location, Bowtie can return all the possible alignments for that sequence read which is required by 'Bowstrap'. 'BowStrap' reads the Bowtie output, and for some selected number of iterations, calculates an RPKM value for each gene model. At each iteration, if a RNA seq read was aligned to more than one possible location, that read is randomly assigned to one of those potential locations. From the set of RPKM values calculated across all Bowstrap iterations, an averages and standard deviations of RPKM are determined for each gene model. A statistical measurement to determine if expression of a gene models has been detected at significant levels is calculated as a Cumulative Normal Distribution (CND) and expressed as a pValue (**Eq. 1**)(1)

Where μ is the average and σ is the standard deviation of a re-sampled gene model's RPKM. As this equation will fail to return a value if the average or standard deviation is equal to 0, if the μ is equal to 0, then the CND pValue is set equal to 1, else if σ is equal to 0, then the CND pValue is set equal to 0. A CND pValue close to zero indicates statistically significant levels of detected gene expression.

In our analysis, all sequence reads were trimmed to 46 bp. Optimal read length for expression analysis is impacted by system and sequence characteristics [55] but the 46 bp read length was empirically determined to yield good sequence alignments to *L. bicolor *and aspen gene models. Bowtie indexes were generated from sets of published JGI gene models for *L. bicolor *and aspen. The default Bowtie conditions were used to generate alignments for all sets of sequence reads to gene models, except for setting Bowtie to return all possible sequence alignments. We used 1000 iterations in 'BowStrap' for the calculation of average and standard deviations of RPKM values. The source code for a PERL implementation of 'BowStrap' is available at [http://www.bio.anl.gov/molecular_and_systems_biology/bowstrap/Bowstrap_download.html]. 'Bowtie' and 'BowStrap' output files are available at the Gene Expression Omnibus (GEO) as series record GSE28157 (http://www.ncbi.nlm.nih.gov/geo/ ).

Analysis of alignments generated with Bowtie indicated a total of 53% of the total sequence reads aligned to *L. bicolor *or aspen gene models. 55.2% of those aligned reads were to *L. bicolor *gene models and 45.8% to aspen gene models. A small fraction of the total aligned reads, 0.49%, aligned to both *L. bicolor *and aspen gene models. Of the entire set of aligned reads, 82% aligned to a single location with the balance aligned to two or more possible locations in the set of gene models. Alignment of reads to the genome scaffolds indicate 57% of the sequence reads aligned to *L. bicolor *or aspen genomic sequence. We observed that 3.2% of sequences aligned to gene models but not to genomic sequence indicating that these sequences align across gene model splice junctions. A fairly high fraction (11.1%) of sequence reads align to genomic sequence but not to gene models. These aligned sequences may indicate the presence of genes that are expressed but have not been identified in the set of gene models or errors in the published gene models. This is consistent with a recently reannotation of the *L. bicolor *genome which increased the number of gene models by ~6% (JGI gene portal). The revised annotation may also account for a fraction of the 36.1% of sequences that align to neither gene models nor genomic sequence. Some of these unaligned reads may come from errors or deviations in the assembled *L. bicolor *and aspen genomic sequences. Some fraction of the unaligned sequence reads likely derive from erroneously called or multiple splice sites in the set of gene models. Previous analysis of deep RNA sequencing in *L. bicolor *have identified that up to 60% of *L. bicolor *gene models contain at least one deviation from expressed gene sequences [56]. The last likely source of un-aligned sequence reads is sequencing errors and the error rates inherent in Illumina sequencing have been previously well characterized [57-59]. A single insertion or deletion in a sequence read will render it un-alignable under the 'Bowtie' criteria used here. More than two substitution errors in a sequence read will also prevent it from finding an alignment. aspen The observed percentage of reads aligned at a high stringency to the gene models is comparable to similar transcriptomic experiments [60-63]. To generate a measurement of the specificity of reads that were found to align to gene models, we generated a synthetic set of reads that were the reverse, but not the reverse compliment, of all observed sequences reads (entire dataset). This synthetic set of reads retains the frequency and relative distribution of nucleotides from the observed data. Aligning the synthetic read set to gene models identifies a total of 28 (0.0001%) aligned reads. Inspection of the aligned synthetic reads indicated that they are comprised of 2 or 3-mer repeats. Alignment with the synthetic read set demonstrates that observed alignments are highly specific and miss-alignments are not expected.

In our transcriptomic data, at a CND-pValue < 0.001, for aspen there are 27318 expressed gene models out of a total possible 45555 (60.0%) gene models. For *L. bicolor*, there are 12856 significantly expressed gene models out of 26014 (62.4%).

### Gene Model Annotation

Annotation to gene model predicted proteins was provided by JGI publicly available downloads using the following files: "Lacc.ecAnnot.txt" for (EC) annotation for *L. bicolor *best gene models, "Lbicolor_GO.txt" for Gene Ontology (GO) annotation for *L. bicolor *best gene models, "mntPop.ec.Annotation.txt" for Enzyme Commission (EC) annotation for aspen gene models, and "Poptr1_1.goinfo.tab" for Gene Ontology (GO) annotation for aspen gene models. The genome annotation resources used in this study were derived by a combination of automated and manual approaches that are described in the Additional material associated with the genome sequence publications.

Using a strategy employed by other investigators [64-66], we used gene models from the JGI sequenced and closely related *P. trichocarpa *as surrogates for the *P. tremulous *genes. There are 12,813 published *P. tremuloides *ESTs in NCBI with an average length of 472 bp, for which the longest sequence is 848 bp and the shortest sequence is 101bp. The limited number of available EST sequences does not represent a complete catalogue of *P. tremuloides *genes and the average sequence length limits functional prediction approaches. To verify that this is a reasonable substitution, we BLAST the *P. tremuloides *ESTs against the JGI predicted best gene models for *P. trichocarpa*. 95% of P. tremuloides ESTs hit JGI *P. trichocarpa *gene models at an e-value threshold of 1e-6. The average percent identity is 93% with a minimum observed 73% identity. 422 (3.3%) P. tremuloides ESTs share 100% identity with *P. trichocarpa *gene models. While the species are not identical and the possibility of presence of unique genes in one species cannot be completely dismissed, the level of similarity observed is suitable for a system-scale analysis of the *P. tremuloides *transcriptome.

10% of *L. bicolor *gene models have EC number annotations and 37% have GO Molecular Function annotation. 13% of aspen gene models have EC number annotations and 41% have GO Molecular Function annotations. Gene models with GO annotations amino acid transport (GO:0006865), Ammonium transporter activity (GO:0008519), Sugar porter activity (GO:0005351), and Inorganic phosphate transporter activity (GO:0005315) are the relevant transporters in the mycorrhizal metabolome.

### Predict mycorrhizal metabolome from transcriptomic data

In this manuscript, we use the term "enzyme function" to describe a specific annotation applied to an enzyme, i.e. "Phosphotransferases with an alcohol group as acceptor". We use "enzyme reactions" to refer to metabolite transformations catalyzed by an enzyme function, i.e "ATP + D-Glycerate ↔ ADP + 3-Phospho-D-glycerate". A unique enzyme function may catalyze more than one enzyme reaction and an enzyme reaction may be catalyzed by more than one unique enzyme function. A metabolite is a molecular compound that is a reactant or product in an enzyme reaction. A network, constructed from annotated unique enzyme functions, enzyme reactions and inferred metabolites, is used to generate the predicted mycorrhizal metabolome. The set of metabolic reactions in KEGG databases was used to represent the set of all possible enzyme reactions in the mycorrhizal metabolome and Enzyme Commission (EC) number annotations for enzyme function for genes models were used to assign unique enzyme functions to the predicted proteins encoded in the mycorrhizal transcriptome. A unique enzyme reaction was included in the mycorrhizal metabolome if a gene annotated to an enzyme function that catalyzed that reaction was detected as significantly expressed in the mycorrhizal transcriptome. All metabolites associated with included enzyme reactions were included in the predicted mycorrhizal metabolome.

### Map genomic and expressed gene models to KEGG metabolic pathways

Annotation of proteins encoded by predicted gene models were obtained from publically available data resource at the Joint Genome Institute (http://www.jgi.doe.gov/) ftp archive. Lists of reactions in the form of reactants, products, and mediating EC enzyme activities were acquired from KEGG (ftp://ftp.genome.jp/pub/kegg/pathway/). Sets of enzyme activities associated with individual KEGG pathways were also collected from the KEGG website. The KEGG database of metabolic interactions includes 1329 organism-specific metabolomes, including 14 complete plant and 51 fungal metabolomes. Of the 378 pathways in KEGG, 27 are plant-specific pathways. The set of plant and fungal metabolomes includes those specific to *P. trichocarpa *and *L. bicolor*. KEGG includes over 8000 individual metabolic reactions, between nearly 15 thousand metabolites and mediated by over 2500 specific EC activities. Every gene model in aspen or *L. bicolor *annotated with a specific EC activity was mapped to KEGG enzymatic reactions as described below.

### Identify enriched annotations and pathways in sets of expressed enzymes

In this analysis, it is required to determine if a particular annotation appears in a subset of genes, relative to the frequency that annotations appear in the superset, at a frequency greater than expected by chance. For this, the Cumulative Binomial Distribution (CBD) was used (**Eq. 2**). The CBD as written here returns the probability that there are at most number *x *successes in n trials where each trial has a probability of success *p*.(2)

Where *x *is equal to the total number of successes in a sample, *n *is equal to the sample size, *p *is equal to the probability of success of each sample, and *Annot *is a specific annotation. For the application here, the number of successes *x *is equal to the number of times a particular annotation *Annot *is present in a subset of gene models and the sample size *n *is the number of gene models in the subset. The probability p is the proportion of gene models in the superset attributed with annotation *Annot*. A CBD pValue close to zero indicates an enrichment of that annotation in the subset while a CBD pValue close to one indicates a depletion of that annotation relative to the distribution of the annotation in a larger set.

Significant enrichment of specific annotations in the sets of expressed mycorrhizal genes, relative to the total number of those specific annotations in the annotated genomes and aspen and *L. bicolor*, was calculated. For the calculation, the number of successes is equal to the number of genes with a specific annotation in the set of expressed gene models. The sample size is the total number of expressed gene models. The probability of success is the frequency of that specific annotation in the complete genome. Specific annotations considered were gene models with an EC activity, sugar transporters, nitrogen transporters, and inorganic phosphate transporters.

KEGG pathways were identified if they were enriched for expressed EC activities in common between the sets of aspen and *L. bicolor *expressed gene models. For each KEGG pathway, the number of successes is equal to the number of EC activities in the set of expressed aspen and *L. bicolor *gene models that map to that pathway. The number of trial is the total number of EC activities expressed in mycorrhizae by aspen and *L. bicolor*. The probability of success is equal to the number of EC activities associated with that KEGG pathway divided by the total number of EC activities expressed by aspen and *L. bicolor*. Specific KEGG pathways were also identified if they were statistically significantly enriched for mapped genes with EC activities unique to expression in either aspen or *L. bicolor*. In this analysis, for each KEGG pathway, the number of successes is equal to the number of gene models in the set of expressed aspen or *L. bicolor *gene models that map to that pathway. The number of samples is equal to the total number of expressed gene models in mycorrhizae by aspen or *L. bicolor*. The probability of success is equal to the number of aspen or *L. bicolor *gene models that map to that KEGG pathway divided by the total number of expressed gene models by aspen or *L. bicolor*. Enriched KEGG pathways were reported at a significance of p < 0.01, and if at least 25% of the genes or activities associated with that KEGG pathway were also in the complete genomic set of aspen or *L. bicolor *gene models.

The complete set of KEGG pathway EC activities was collected from ftp://ftp.genome.jp/pub/kegg/pathway/ec/. Identifying expressed activities and transported small molecules in KEGG global metabolism was performed using the KEGG PATHWAY Query tool.

## Abbreviations

(ECM): Ectomycorrhizal; (NGS): Next Generation Sequencing; (JGI): Joint Genome Institute; (GO): Gene Ontology; (EC): Enzyme Commission; (KEGG): Kyoto Encyclopedia of Genes and Genomes; (WPM): Woody Plant Media; (RPKM): Reads Per Killobase per M RNAseq reads; (CBD): Cumulative Binomial Distribution; (CND): Cumulative Normal Distribution.

## Authors' contributions

Conceived and designed the experiments: FC, GP, PL. Performed the experiments: AS, GT, PL. Analyzed the data: FC, GP, PL. Contributed reagents/materials/analysis tools: LC, PL. Wrote the paper: FC, LC, PL All authors read and approved the final manuscript

## Supplementary Material

Additional file 1 **Data S1. The complete predicted mycorhhizal matabolome is available for download as a 'Cytoscape' file. Figure S1**. An interactive version of Figure 1 is available at http://www.bio.anl.gov/molecular_and_systems_biology/MycorMetabolome/Supplementalfigures.htm. Click here for file
